# Southern elephant seals (*Mirounga leonina* Linn.) depredate toothfish longlines in the midnight zone

**DOI:** 10.1371/journal.pone.0172396

**Published:** 2017-02-24

**Authors:** John van den Hoff, Robbie Kilpatrick, Dirk Welsford

**Affiliations:** Australian Antarctic Division, Kingston, Tasmania, Australia; Stockholms Universitet, SWEDEN

## Abstract

Humans have devised fishing technologies that compete with marine predators for fish resources world-wide. One such fishery for the Patagonian toothfish (*Dissostichus eleginoides*) has developed interactions with a range of predators, some of which are marine mammals capable of diving to extreme depths for extended periods. A deep-sea camera system deployed within a toothfish fishery operating in the Southern Ocean acquired the first-ever video footage of an extreme-diver, the southern elephant seal (*Mirounga leonina*), depredating catch from longlines set at depths in excess of 1000m. The interactions recorded were non-lethal, however independent fisheries observer reports confirm elephant seal-longline interactions can be lethal. The seals behaviour of depredating catch at depth during the line soak-period differs to other surface-breathing species and thus presents a unique challenge to mitigate their by-catch. Deployments of deep-sea cameras on exploratory fishing gear prior to licencing and permit approvals would gather valuable information regarding the nature of interactions between deep diving/dwelling marine species and longline fisheries operating at bathypelagic depths. Furthermore, the positive identification by sex and age class of species interacting with commercial fisheries would assist in formulating management plans and mitigation strategies founded on species-specific life-history strategies.

## Introduction

Deep oceanic waters beyond the photic zone define the Earths’ largest biome [1]. The bathypelagic zone or so-called “midnight zone” between depths of 1000 and 4000m is typified by extreme hydrostatic pressures, low dissolved oxygen content, cold water-temperatures and complete darkness yet a very large biomass of forage species exist there [2]. The sheer depths at which these organisms exist make them available to a select group of air-breathing predators; only the elephant seals (*Mirounga* spp. Phocidae) and some toothed whale (Odontocete) species (*e*.*g*. Ziphiidae and Physeteridae) have evolved the ability to dive and forage beyond 1000m [3]. Over a much shorter time interval (< 200 years) shallow diving modern humans (*Homo sapiens* Hominidae) have devised technologies capable of exploiting those same biological resources [4]. In that short time interval increased human capabilities combined with escalating population demands, poor industry management and illegal fishing operations have contributed to overfishing and alterations of deep-sea marine ecosystems globally [5]. Deep foraging predators no longer have exclusive access to the biological resources available in the deep oceans and furthermore as future fisheries develop we might expect new and unforseen conflicts to arise.

Marine mammal—fisheries interactions take many forms but catch depredation (*i*.*e*. the direct removal or damage to catch or bait) and by-catch (*i*.*e*. the incidental take of non-target species, individuals of which can be released unharmed or injured, or killed) are of greatest concern worldwide [6–8]. Injuries and deaths result when individuals become either entangled or hooked by fishing gear, or when fishermen take retaliatory action to protect their catch [7]. Depredation is of economic concern to a fishery because of losses associated with gear damage, the removal or mutilation of catch and catch reductions due to bait removal. By-catch of charismatic megafauna is of economic concern because of the potential for negative public perceptions and the generation of restrictive regulations on the fishery [9]. Depredation may also provide predators with a source of food that they otherwise would not have access to, thereby changing food web dynamics. Moreover unchecked indiscriminate by-catch can have far ranging ecological impacts including trophic restructuring [10] and non-target species population declines [6].

Coastal and shallow water longline fisheries are exposed to depredation by shallow-diving marine mammals [7] while the deeper-water demersal longline fisheries are at risk from both shallow and deep diving species especially when catch is exposed during gear retrieval. One fishery receiving such unwanted attentions is the Patagonian toothfish (*Dissostichus eleginoides* Notothenidae) longline fishery targeting fish around Southern Ocean seamounts, sub-Antarctic islands and southern South America [11, 12]. Sperm whales (*Physeter macrocephalus* Physeteridae) and killer whales (*Orcinus orca* Delphinidae) are reported to selectively remove toothfish potentially reducing catch rates by up to 30% compared with lines hauled in their absence [12]. Other studies have shown the Antarctic fur seal (*Arctocephalus gazella* Otariidae), colossal squid (*Mesonychoteuthis hamiltoni* Cranchiidae), sleeper shark (*Somniosus* ssp. Somniosidae) and porbeagle shark (*Lamna nasus* Lamnidae) also depredate toothfish fisheries ([12–14], AAD unpublished data).

Green et al. [15] identified the potential for interactions between southern elephant seals (*Mirounga leonina*) and the toothfish industry operating within Australian exclusive economic zones (EEZ) surrounding Macquarie Island and the Heard and the McDonald Islands. However that concern was based primarily upon the perception that a trawl fishery would compete for food resources, rather than directly interacting with the seals. Fishery dependent monitoring subsequently recorded rare by-catches of elephant seals, including decomposed corpses that were unlikely to have drowned as a result of the trawl fishery. As the toothfish fisheries developed and came to rely more heavily on longlining, reports of entanglements and hooking’s became more frequent [16].

With the Australian toothfish fisheries becoming increasingly dominated by longline fishing, there is a general need to investigate the nature and extent of interactions between the fishery, its methods and bathypelagic species that target those same resources. Such data and observations are difficult to gather because toothfish fisheries operate in remote geographic locations and at extreme depths during the austral winter. However, since their commencement Australian fisheries have benefited from comprehensive accredited observer coverage and recent deployments of a deep-sea video camera system attached to working longlines. On the basis of newly acquired video footage we provide the first-ever direct evidence of elephant seals depredating Patagonian toothfish from longlines set in deep water. We discuss a potential mitigation response based on the seals’ life-history strategy.

## Materials and methods

### Study area

**Fig 1:**
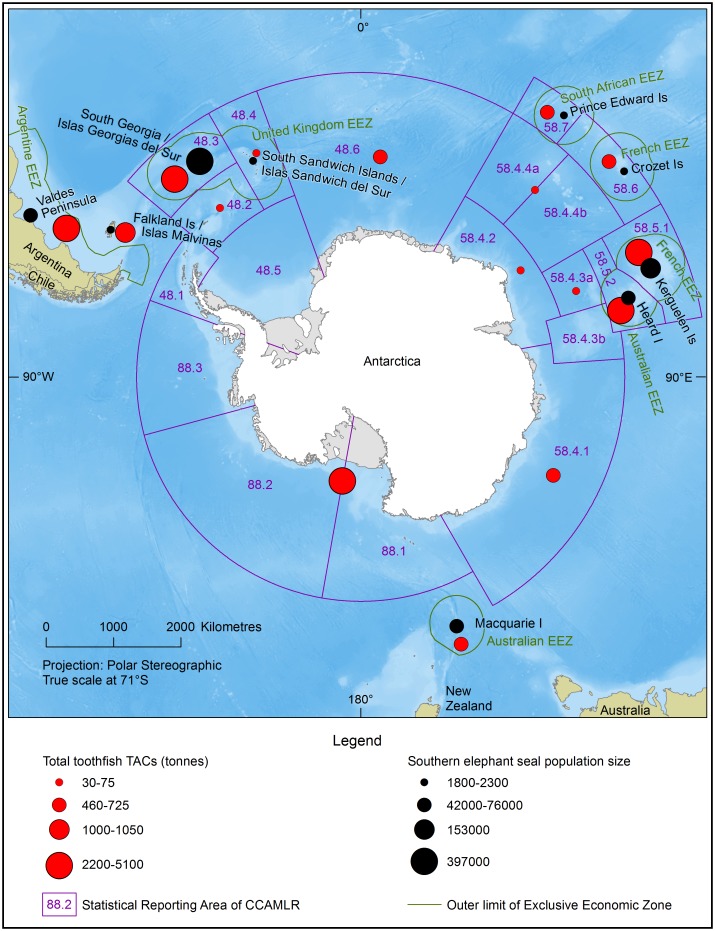
Total Allowable Catches (TAC’s) for Patagonian toothfish (*Dissostichus eleginoides*) and population estimates for the southern elephant seal (*Mirounga leonina*). Southern Hemisphere polar projection showing the Commission for the Conservation of Antarctic Marine Living Resources (CCAMLR) boundaries, national Exclusive Economic Zones (EEZ’s), TAC’s for Patagonian toothfish within CCAMLR regions (red bubbles), southern elephant seal population estimates (black bubbles) and ocean bathymetry (blue shading; light = shallow). Figure produced by the Australian Antarctic Data Centre (c) Commonwealth of Australia 2016. Bathymetry: GEBCO (General Bathymetric Chart of the Oceans) 2014 grid, version 20150318. Coastline: Antarctic Digital Database version 6 (Antarctica), DeLorme, ESRI (other coastlines).

With an estimated area of 2 x 10^6^ km^2^ the volcanically active Kerguelen Plateau (KP) is the world’s second largest submarine plateau [17]. Emerging above the Southern Ocean over the KP are the French Îles Kerguelen (IK) and the Australian Heard and McDonald Islands (HIMI) external territories. France and Australia each administer a 200 nautical mile marine EEZ around their respective islands and within which commercial fisheries target Patagonian toothfish (Fig 1). Both EEZ’s lay within areas covered by the Convention for the Conservation of Antarctic Marine Living Resources (CCAMLR), with the Australian EEZ mostly falling within CCAMLR Statistical Division 58.5.2 (Fig 1). Under the Antarctic Marine Living Resources Conservation Act (1981), adopted conservation measures are given effect under Australian law and include regulations on gear type, catch limits and season closures established to mitigate seabird by-catch (https://www.ccamlr.org; accessed 03/02/15).

### Patagonian toothfish longline fisheries within the study area

Combined, CCAMLR areas 58.5.1 and 58.5.2 support the largest total allowable catches (TAC’s) of toothfish for any region in the world (Fig 1). Longline fishing began in the IK-EEZ during 1991 and a decade later (2002/03) in the HIMI-EEZ; both fisheries continue to date. Toothfish are generally targeted by longlines with squid baited hooks set at depths between 800 and 2000m but fishing on the KP has occurred at all depths between 300 and 2500m [18, 19]. Fishing is prohibited in HIMI-EEZ marine reserve, and in waters shallower than 500m within the IK-EEZ [20].

Demersal longline vessels licensed to fish in the HIMI and IK EEZ’s were similarly equipped autoliners using a weighted-line system to mitigate seabird by-catch [21]. Up to five longline vessels operate within the HIMI-EEZ during each fishing season (1 May to 14 September). Further longline fishing is permitted during season extension periods (15 April to 30 April and 15 September to 14 November) provided the permit holder has written agreement from AFMA (http://www.afma.gov.au/fisheries-services/concession-holders-conditions). The KI-EEZ fishery opens to 6–7 vessels year-round with the exception of 1 February to 1 March or 15 March [11]. Approximately 20–30 million baited hooks are deployed per annum in the IK and up to 15 million in the HIMI.

### Southern elephant seals within the study area

A combined estimate of 215,000 southern elephant seals (*ca*. 30% of the worlds’ population, [22]) breeds on Îles Kerguelen and Heard Island (Fig 1). Telemetry of seals from those locations showed a proportion of tracked adult seals remained on the KP foraging close to the seafloor [23–26] while the remainder travelled into the open ocean and Antarctic waters. Most foraging migrations from December to April occurred over the KP between IK and HI in waters < 1000m deep and those juveniles spent up to 90% of their time on the KP shelf diving predominantly (~58–70%) to the sea floor at depths between 400 and 700m [24, 25]. Juveniles also used the KP more during January to May (85%) compared with the later winter months (15%), as might be expected from their haulout pattern [23]. Adult female seals tended to travel beyond the KP to forage in open ocean frontal waters, at the marginal ice zone and over the Antarctic continental shelf but some remained on the KP [23–26].

### Direct observations of southern elephant seals

Kilpatrick et al. [27] designed a compact autonomous deep-sea video camera system that could be deployed from commercial autoline fishing vessels. Over a period of four years, 53 camera deployments were made in the HIMI-EEZ gathering over 200 hours of footage that captured 10 minutes of footage of three seals interacting with longlines at the sea floor. The camera system included two 500 lumen LED lamps capable illuminating 3–6m of seafloor. Therefore one caveat over any observations described herein is the possibility that the inbuilt light source attracted the seals to the camera and longline. While we cannot absolutely discount that possibility there is evidence to suggest southern elephant seals interact with longlines in the absence of external light sources ([28], S1 Table). The water depth at each camera deployment was determined from the fishing vessels depth sounder and all observation times were recorded in UTC (local HIMI-EEZ time is UTC +5 hours).

No permits were required to deploy the cameras used for this study as they were attached to actively fishing longlines set from Australian Fisheries Management Authority (AFMA) licensed vessels with statutory fishing rights (http://www.afma.gov.au/fisheries-services/concession-holders-conditions).

### Fisheries interactions

Catch variables such as fish weight, fishing effort and by-catch were reported to the AFMA by accredited observers and vessel masters to meet a requirement of the conservation measures enforced upon commercial fisheries operating within CCAMLR convention areas. Observers and masters are also required to report interactions with marine mammals and seabirds to AFMA within 24 hours of such an interaction occurring. The AFMA data were summarised (S1 Table) to categorise interaction types and to determine temporal and spatial variability in southern elephant seal by-catch.

Without appropriate experience, classification of marine mammals by sex and age class is difficult. For example, juvenile male elephant seals can be mistaken for juvenile/adult female seals but the adult male seal is more easily recognisable. For this reason we did not assign a specific sex and age to each elephant seal interaction reported to AFMA for the toothfish long-line fishery.

### Statistical analyses

Patterns of elephant seal interactions and fishing effort by year and month of fishing activity (2003–2015) were analysed using Generalised Additive Models (GAM’s) within the *mgcv* package of R [29–31]. The mean and variance of mortalities.month^-1^ (0.37 and 0.50, respectively) were sufficiently similar indicating that the data was not over-dispersed. Month and year were included as smoothing terms, with log(hooks) set as an offset so the model output could be interpreted as a ‘catch rate’ of seals per unit hooks. Although the data were not over dispersed we used a negative binomial error structure because this approach is considered more robust than poisson or quasipoisson distributions when analysing zero-inflated distributions [32].

## Results

Kilpatrick et al’s [27] video camera system recorded the activities of three adult (7+ years of age) male southern elephant seals (identified by J.v.d.H) as they interacted with commercial longlines actively fishing for Patagonian toothfish at depths in excess of 1000m.

### Direct observations

Seal #202 was observed on the 02/May/2011 briefly passing the camera deployed at 1660m, the deepest deployment for this study (S1 Video). No hooked fish were visible within the cameras’ field-of-view.

Seal #209 was observed approaching a live hook-caught toothfish at a depth of 1030m on the 07/July/2010. The seal made an unsuccessful attack on the fish (S2 Video at 00:16:43, Fig 2A); the fish evading capture by swimming vigorously upward through the water column as far as the long-lines’ snood allowed (*ca*. 450mm). Seal #209 then returned to successfully grasp the same fish thereby causing a section of long-line to be lifted from the seafloor (S2 Video at 00:17:19). The line returned to the seafloor with a sharp jerk, and later footage recorded as the water column cleared showed the seal had depredated the longline.

**Fig 2:**
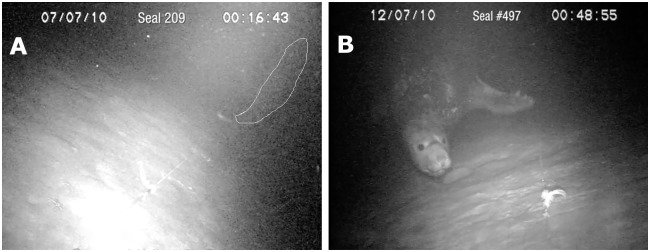
Direct evidence male southern elephant seals (*Mirounga leonina*) interact with Patagonian toothfish (*Dissostichus eleginoides*) caught on longlines deployed in deep water. (A) Seal #209 unsuccessfully attacking a hook-caught toothfish. The seal is outlined in white and the fish is directly in front of the seal. The longline can be seen in the foreground resting on the seafloor. View S2 Video for a full appreciation of the interaction. (B) Seal #497 approaching the underwater video camera system. Note: the seal appears aware of the camera systems presence, its eyes are open and vibrissae erect. The longline can be seen resting on the seafloor near the seal’s left fore-flipper. View S3 Video for a full appreciation of the interaction.

Seal #497 provided the longest observational record on the 12/July/2010. This male seal was observed at a depth of 1273m swimming along the longline toward the camera (S3 Video). The seals’ eyes were open and his vibrissae extended, the eye direction and the approach behaviour showed that the seal was aware of the camera (Fig 2B). The seal bumped the camera, then turned and swam back along the long-line to attack a hook-caught toothfish. The seal lifted the fish from the seafloor but dropped it a short time later only to attack another hook-caught fish or baited hook beyond the limit of the camera’s illuminated field-of-view, this time lifting the long-line from the seafloor. At 00:48:30 (S3 Video) the camera was bumped from behind and a seal we presume from its age and markings to be seal #497 re-inspected the hook-caught fish before disappearing from view at 00:51:41; a period of between six and seven minutes had elapsed.

### Fisheries reported interactions

Twenty-nine (29) southern elephant seals of unconfirmed age and sex were reported interacting with the Australian longline fishery operating legally within the HIMI-EEZ from 2003 to 2015 (S1 Table); six seals were hooked and 23 became entangled in some part of the longline. The majority (26 or 89%) of the seals hauled to the surface were dead (drowned).

Comparison of the Akaike’s Information Criterion (AIC) scores between a model of the form *number of mortalities ~ s(year) + s(month)* (df = 7.9, AIC = 1456) was superior to the model *number of mortalities* ~ *s(year)* (df = 4.9, AIC = 1556), indicating that both month and year were significant in explaining the number of mortalities reported (S1 Fig, S2 Table). Mortalities were more probable in the middle months (austral winter) of the fishing season. Mortalities declined between 2003 and 2005, then remained fairly stable before slowly increasing since 2012 (S1 Fig).

### Spatial and temporal overlap

**Fig 3:**
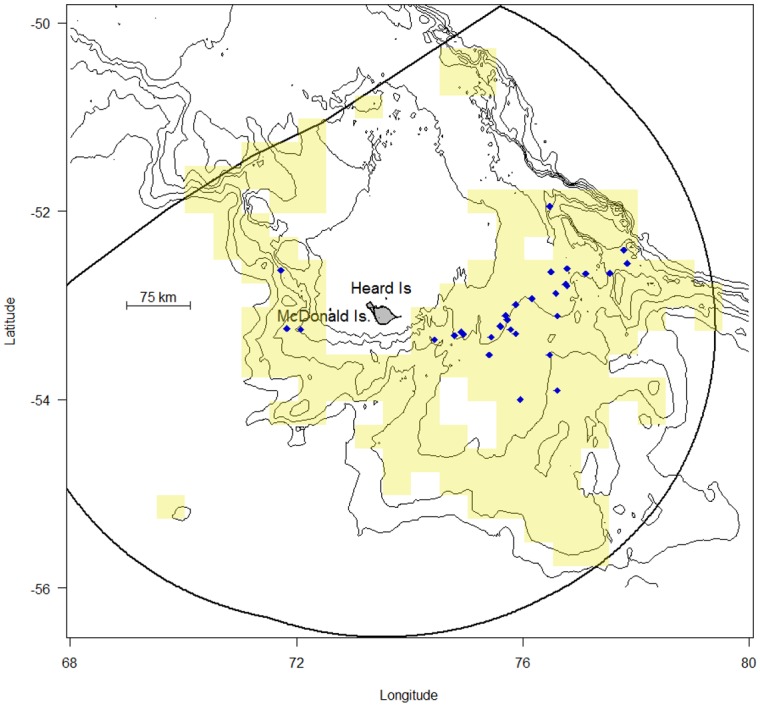
Locations of interactions between southern elephant seals (*Mirounga leonina*) and the Patagonian toothfish (*Dissostichus eleginoides*) longline fishing industry operating within the Australian EEZ at Heard Island and the McDonald Island (HIMI-EEZ), 2003–2015. Most interactions occurred at depths near the 1000m isobath. Cream 0.5° x 0.25° grids indicate where longline fishing has occurred but do not reflect fishing effort. Blue points are the midpoint of longline sets were a seal interaction has been reported (S1 Table). Heavy black line is the HIMI-EEZ boundary; thin black lines are isobaths at 500m intervals [33]; land is shown as grey.

It was not possible to overlay the spatial distribution of fishing effort for HIMI fishing vessels with at-sea locations for free-living adult male southern elephant seals because: a) very little telemetry data exists for adult male southern elephant seals from KP breeding sites (n = 5, [23]), b) data gathered for juvenile seals and adult females are unlikely to accurately represent the foraging preferences for adult males, and c) exact fishing locations and effort are commercially-in-confidence. We were however able to plot the general distribution of fishing fleet activities with the locations of southern elephant seal interactions reported to the AFMA (Fig 3).

The spatial distribution of the fishery within the HIMI-EEZ indicates the majority of waters between 500 and 2000m depth were fished (Fig 3). The 29 reported interactions with southern elephant seals were mostly along the 1000m isobath (range 791–1607m, mean = 1055m) and most frequent to the east of Heard Island (Fig 3). The three adult male southern elephant seals we recorded on the deep-water camera system were observed at water depths >1000m to the west of Heard Island.

**Fig 4:**
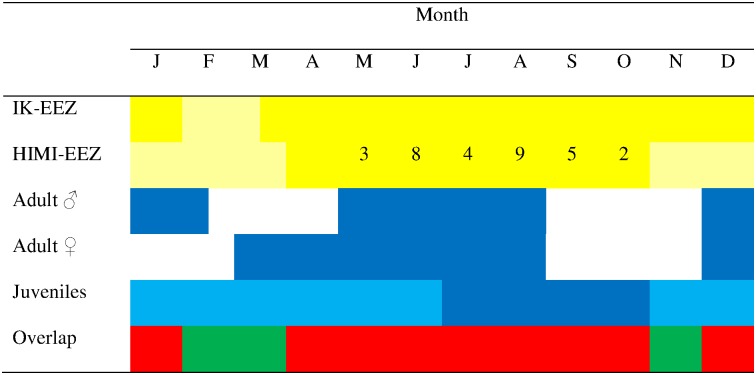
Time lines for commercial fisheries targeting Patagonian toothfish (*Dissostichus eleginoides*) on the Kerguelen Plateau and the at-sea phases, by sex and age class, for southern elephant seals (*Mirounga leonina*) breeding on Isles Kerguelen (IK) and Heard Island (HI). Dark yellow = fishery open and active (numbers within the HIMI-EEZ boxes are total southern elephant seal interactions in that month, 2003–2015. Light yellow = IK-EEZ closed to fishing and HIMI fishery inactive. Light blue = a proportion of the juvenile population is on land. White = adult seals ashore breeding or moulting. Green/Red = low/high potential for overlap between the seals and KP fisheries.

Temporal overlap between the HIMI and IK fisheries and the seals was greatest during the austral winter months from May to September (Fig 4). This is within the period when adult seals are foraging at sea following their breeding and moult fasting periods.

## Discussion

Deployments of an autonomous deep-sea camera system [27] have for the first time provided unequivocal evidence that adult male southern elephant seals (*M*. *leonina*) depredate Patagonian toothfish (*D*. *eleginoides*) from actively fishing longlines. Unlike sperm whales and killer whales that depredated toothfish longlines at shallow depths during catch retrieval [34] the seals depredated toothfish from actively fishing longlines set at depths beyond 1000m. This novel finding further highlights how little is known about the suite of species which interact with commercial fisheries beyond shallow depths. Information gathered using deep-sea camera systems attached to fishing gear could provide a means with which to discover such cryptic interactions and would also assist with the development and implementation of appropriate mitigation strategies.

### Spatial overlap between the fishery and the seals

Quantifying horizontal overlap between the Patagonian toothfish fishery and adult male southern elephant seals in this study was thwarted principally because of the lack of available telemetry data for adult male southern elephant seals migrating from IK and HI, a situation that requires rectification in the light of our findings. Given the three seals we recorded were adult males, the only five adult male seals ever tracked from HI dived to around 1000m over the KP [23], and the majority of longline fishing effort occurred at depths between 800 and 1600m (Fig 3, S2 Fig) we find adult male seals are the demographic category most, but not exclusively, at risk of incidental mortality in this fishery. Telemetry studies [25, 26] and observer reports (S1 Table) suggest juvenile seals and adult females may also interact with the fishery at depths between 400 and 700m. However the young and adult females generally dispersed beyond the KP into the open ocean to feed. Maintaining or improving separation between the fishery and both the juvenile age classes and the adult females is an important objective because removals from those demographic classes are likely to have a negative impact upon the seals’ population trajectories.

### Depredation of toothfish longlines: Signs and mitigation

Quantifying depredation is not a straightforward process [11], yet its consequences can have high economic and ecological costs as well as being an important variable in fish stock assessments [12, 26]. The majority of depredation reported previously for this multinational fishery occurs during longline retrieval when sperm and killer whales typically depredate hook-caught toothfish near the sea surface [34, 35]. However, Purves et al. [35] also noted that only toothfish lips were present on 13% of observation hooks when no marine mammals were in sight; an observation that led them to surmise other deep-diving cetaceans or bathypelagic predators such as the sleeper shark (*S*. *pacificus*) could be implicated.

The large canine teeth (tusks) of the adult male elephant seal [36] have similarities with toothed whale species. We think the seals dentition, combined with the considerable effort required to lift a hooked fish attached to a weighted longline, could leave similar markings and fish remains as those left by toothed whales [34]. Therefore an undetermined proportion of depredation observed within the fishery, and previously attributed to toothed whales, may have been caused by elephant seals.

Depredation of actively fishing longlines set at the sea floor circumvents the majority of physical depredation mitigation devices (PDMD) currently used to protect catch from marine mammal depredation. The most effective of those measures, halting longline retrieval and moving the fishing vessel away from the area [8, 12] would appear ineffectual in this case. Such an approach is unlikely to mitigate seal bycatch because the interactions have already taken place cryptically during the line set-period not during line retrieval.

Acoustic harassment devices (AHD) apparently have limited promise as a general marine mammal deterrent in the Southern Ocean pelagic longline fisheries [12], and diving elephant seals showed little response to acoustic stimuli [37]. This evidence suggests AHD effectiveness is probably of limited value for deterring elephant seals from longlines. However AHD technologies continue to develop and could be trialled in the toothfish fishery because target-specific deterrents have shown some recent potential [38].

The KP fishing season is operational at the exact time adult seals are at sea replenishing their fat reserves after fasting during their breeding and moulting phases (Fig 4). Adult male seals potentially dive deepest and often forage benthically at depths in excess of 1000m [23] thereby elevating the risk of interaction with the toothfish longline fishery compared with the juvenile and adult female age classes. Potentially lethal interactions between breeding aged seals and the fishery could perhaps be minimised by rescheduling the fishing season to March-May and September-November when adult seals are ashore (Fig 4).

Changing the seasonality of the HIMI fishery would of course need to be considered in the light of its effects on by-catch of other species such as seabirds and the commercial viability of the fishery. Presently the HIMI fishery operates within a ‘core’ season (1 May to 14 September), a measure designed to reduce seabird mortality as the fishery operates outside critical seabird breeding seasons. Confining fishing to March-May and September-November could ensure by-catch of seals and seabirds is reduced or remains at stable low levels but it is not presently known what the implications may be in a commercial sense.

### By-catch and incidental mortality

None of the southern elephant seals filmed during this study became entangled or hooked on longlines, yet 29 entanglements and hookings were reported for the Australian fishery (S1 Table). The majority of seals seen at the surface (n = 26) were dead (drowned), most likely because the line soak period (24–48 hours) exceeds the dive duration capabilities of elephant seals (*ca*. 2 hours [39]). Seals seen alive at the surface suggests those animals interacted with the longline close to line retrieval or during line retrieval itself.

While elephant seal interactions were reported in the Australian fishery, no such interactions have been reported for the nearby IK fishery over a fishing similar period. This is a curious result given the close proximity and similarities in fishing methods (observer coverage, bait type, depth and season) between the two fisheries, but there may be several explanations (or a combination of explanations) for this apparent anomaly. Firstly, regardless of sex and age class, the majority of elephant seals tracked from HI and IK did not forage over the northern KP where IK fishery operates [26, 40]. And/or secondly, the presence of orca and sperm whales in the IK fishery might have influenced those statistics. Both whale species have been reported attending longline operations within the IK-EEZ but only sperm whales have been recorded in the HIMI-EEZ to date. While sperm whales are largely fish and squid eaters, orca attack and eat southern elephant seals; even large males are potential prey [41]. The seals may therefore actively avoid feeding in areas overlapping orca presence.

Since submission of this manuscript, fisheries observers have reported further incidents concerning southern elephant seal—toothfish longline interactions within the HIMI (AFMA unpublished data). Clearly, the data presented herein and the recent AFMA data are indicative of persistent interactions between the seals and this fishery. We therefore recommend that comprehensive data collection on the number of these interactions is introduced and maintained across all fisheries, and broadened to include additional data. In particular, accurate data on the sex and life stage of the seals involved should be collected so that the demographic impact of these interactions can be assessed across all southern elephant seal populations and mitigation developed if interactions increase especially with female seals.

## Supporting information

S1 Table Minimalized fisheries observer data. Source: Australian Fisheries Management Authority (www.afma.gov.au).
(PDF)

S2 Table Results of generalised additive modelling of southern elephant seal (*Mirounga leonina*) mortalities within the Patagonian toothfish fishery operating within the Heard Island and McDonald Islands Exclusive Economic Zone (HIMI-EEZ) 2003 to 2015. (PDF)

S1 Video Footage of southern elephant seal #202 swimming along a Patagonian toothfish longline set within the Heard Island and McDonald Islands Exclusive Economic Zone at 1660m depth, May 2011. (MOV)

S2 Video Footage of southern elephant seal #209 depredating Patagonian toothfish from a longline set within the Heard Island and McDonald Islands Exclusive Economic Zone at 1030m depth, July 2010. (MOV)

S3 Video Footage of southern elephant seal #497 depredating a Patagonian toothfish longline set within the Heard Island and McDonald Islands Exclusive Economic Zone at 1273m depth, July 2010. (MOV)

S1 Fig Numbers of southern elephant seal (*Mirounga leonina*) mortalities in the Heard Island and McDonald Islands Patagonian toothfish fishery have increased over time, being most common during the austral winter months. Predicted mortalities per 10^−6^ longline hooks set by year (upper panel) and by month (lower panel) from a generalised additive model of data reported for longline vessels operating within the Heard Island and McDonald Islands Exclusive Economic Zone (HIMI-EEZ, data from S1 Table), 2003 to 2015. Plots show the prediction when variables were fixed at a representative value (July for the year effect, and 2009 for the month effect). Dotted lines are 95% confidence intervals.
(DOCX)

S2 Fig Depth distribution for Patagonian toothfish (*Dissostichus eleginoides*) longline fishing effort (hooks set) and three male southern elephant seals (*Mirounga leonina*) seen interacting with toothfish longlines within the Heard Island McDonald Islands Exclusive Economic Zone (HIMI-EEZ). Blue dotted lines show the depths at which the southern elephant seal interactions were recorded.
(PDF)
