# Supplementary material for: Southern elephant seals (Mirounga leonina Linn.) depredate toothfish longlines in the midnight zone
Source: PLoS One. 2017 Feb 24;12(2):e0172396. doi: 10.1371/journal.pone.0172396 (PMC5325274; doi:10.1371/journal.pone.0172396)
Supplement: S1 Table — Source: Australian Fisheries Management Authority (www.afma.gov.au). (PDF) [file pone.0172396.s001.pdf]

S1 Table. Minimalized fisheries observer data. Source: Australian Fisheries Management Authority (www.afma.gov.au). (PDF) van den Hoff et al.

Date	Area	Vessel	Fishing Depth (m)	Qty	Wildlife Species	Dead	Alive	Comments
06-Jun-03	HIMI	7	824	1	Southern Elephant Seal	1		seal became entangled in the backbone and presumably drowned. No other seal sightings.
09-Jun-03	HIMI	7	1106	1	Southern Elephant Seal	1		Approx. 2 + metres in length and an est. 300+ kg, possibly male. Somehow seal got part of the backbone looped around it's neck and was dead when brought to the surface. As this is the second elephant seal to be caught in this area vessel chose to move on
11-Jun-03	HIMI	7	1111	1	Southern Elephant Seal	1		the seal became entangled in the backbone of the longline and was brought to the surface dead. It had presumably drowned.
16-Jun-04	HIMI	7	791	1	Southern Elephant Seal	1		Seal became entangled in the backbone of the line and was brought to the surface dead, presumably drowned. Estimated weight 1.2 tonnes, sex unknown. A snood and hook were trailing from the corner of the seal's mouth.
01-Jul-05	HIMI	4	803	1	Southern Elephant Seal	1		Seal was hooked in the mouth and was dead when reaching the surface. Carcass came off the hook at the surface.
20-Aug-05	HIMI	4	905	1	Southern Elephant Seal	1		Juvenile male seal weighing approx. 500kgs came up entangled in line.
26-Aug-05	HIMI	4	1050	1	Southern Elephant Seal	1		Juvenile male seal weighing approx. 700kgs came up entangled in line.
18-Aug-07	HIMI	7	1607	1	Southern Elephant Seal	1		seal tangled in line. Approx 900kg 2-3 metres in length. Pulled free of tangle when brought to the surface. May have been dead for some time as significant sea lice damage.
13-Sep-08	HIMI	3	811 - 995	1	Southern Elephant Seal	1		Female weighing around 200 kgs was hooked and found entangled on the line
14-Sep-08	HIMI	7	1053	1	Southern Elephant Seal	1		Male weighing approx 2000 kgs was found entangled in the line
03-Jun-09	HIMI	3	1030	1	Southern Elephant Seal	1		Female weighing approx 200 kgs was found dead after being entangled in the mainline. It was not landed but photos were taken.
11-Jun-09	HIMI	3	1099	1	Southern Elephant Seal	1		Seal weighing approx 200 kgs was found dead after being entangled in the mainline most likely while taking a bait
09-Aug-10	HIMI	3	1059	1	Southern Elephant Seal		1	Seal estimated to weigh 200 kgs was hauled up on the longline and was seen to fall off the line when it broke the surface. The seal swam away 'very much alive'. The incident was reported by the first mate as the observer was below doing other duties.
13-May-12	HIMI	1	836	1	Southern Elephant Seal		1	Seal was lightly hooked in the lip as it broke the surface. It gave a vigorous flick of its head and the hook came out of the mouth. It same away in no distress.
14-May-12	HIMI	1	1342	1	Southern Elephant Seal		1	Seal became slightly entangled in the longline when it broke the surface. It rolled away and swam off vigorously.
05-Jul-12	HIMI	3		1	Southern Elephant Seal	1		Female seal was hooked in mouth and wrapped around the mainline. Given observed state of rigor it is believed the seal was caught during the haul. It untangled when reaching the surface. No photos taken.
16-May-13	HIMI	3	1259	1	Southern Elephant Seal	1		Seal weighing around 900 kgs was killed when becoming entangled in the mainline with the line wrapped several times round the animals neck. It was cut free. Photos were taken.
08-Jun-13	HIMI	6		1	Southern Elephant Seal	1		Female seal, approx 2.5 metres in length, was killed when becoming entangled in the mainline. It was not brought onboard. No photos taken.
02-Aug-13	HIMI	6		1	Southern Elephant Seal	1		Female seal, approx 2.5 metres in length, was killed when becoming entangled in the mainline. It was not brought onboard.
10-Aug-13	HIMI	6		1	Southern Elephant Seal	1		Female seal approximately 2.5 metres in length was killed when becoming entangled in the mainline. It was cut free.
21-Aug-13	HIMI	6		1	Southern Elephant Seal	1		Female seal approximately 2.5 metres in length was killed when becoming entangled in the backbone of the line. It was cut free.
22-Aug-13	HIMI	6		1	Southern Elephant Seal	1		Female Seal became entangled in backbone of the line whilst hauling. The animal cut free.
09-Jul-14	HIMI	6		1	Southern Elephant Seal	1		During hauling a female Southern Elephant Seal (approx. 2.5m) was hauled entangled in the backbone of the line. The animal appeared to be deceased and was released from the line. The AFMA observer witnessed the incident.
19-Aug-14	HIMI	6		1	Southern Elephant Seal	1		During hauling a female Southern Elephant Seal (approx. 2.5m) was hauled entangled in the backbone of the line. The animal appeared to be deceased and was released from the line. The CapFish observer witnessed the incident. AFMA observer reported that he believed that the seal took a fish on the line and became hooked, tangled and drowned.
08-Jun-15	HIMI	2		1	Southern Elephant Seal	1		Female Southern Elephant seal of approximately 500-600kgs, was hauled up tangled by its tail in the line. The mammal had drowned but did not appear to have been dead for long. The carcass was not retained.
08-Jul-15	HIMI	3		1	Southern Elephant Seal	1		Southern Elephant Seal came up on the line with the main line tangled around its nose. The seal broke free when it got to the surface but it was believed the mammal was dead.
01-Sep-15	HIMI	6		1	Southern Elephant Seal	1		Southern Elephant Seal came up on the line tangled with the main line. The seal broke free when it got to the surface in the swell but it was deceased.
06-Oct-15	HIMI	5		1	Southern Elephant Seal	1		The seal was hauled up hooked in the mouth (caught on the upper jaw). There was a 5-6kg skate on the hook inside the mouth of the seal.
23-Oct-15	HIMI	5		1	Southern Elephant Seal	1		The seal had been tangled (not clear if hooked) around the tailfin. When the tailfin came out of the water the wraps of line came off and the seal sank very fast (unclear if dead or alive).
