# Supplementary material for: Southern elephant seals (Mirounga leonina Linn.) depredate toothfish longlines in the midnight zone
Source: PLoS One. 2017 Feb 24;12(2):e0172396. doi: 10.1371/journal.pone.0172396 (PMC5325274; doi:10.1371/journal.pone.0172396)
Supplement: S2 Table — (PDF) [file pone.0172396.s002.pdf]

S2 Table. Results of generalised additive modelling of southern elephant seal (*Mirounga leonina*) mortalities within the Patagonian toothfish fishery operating within the Heard Island and McDonald Islands Exclusive Economic Zone (HIMI-EEZ) 2003 to 2015.

**Parametric Term**		**Estimate**	**Std. Error**	***z* value**	***Pr*(>|*z*|)**
(Intercept)		-1.65	0.12	-13.56	<0.01
Smooth Terms	*edf*			X^2	*p*
Month	4.8			120.4	<0.01
Year	4.8			74.8	<0.01
				R^2 (adjusted)	Deviance explained
				0.133	25.3%
