# Supplementary material for: Southern elephant seals (Mirounga leonina Linn.) depredate toothfish longlines in the midnight zone
Source: PLoS One. 2017 Feb 24;12(2):e0172396. doi: 10.1371/journal.pone.0172396 (PMC5325274; doi:10.1371/journal.pone.0172396)
Supplement: S1 Fig — Predicted mortalities per 10−6 longline hooks set by year (upper panel) and by month (lower panel) from a generalised additive model of data reported for longline vessels operating within the Heard Island and McDonald Islands Exclusive Economic Zone (HIMI-EEZ, data from S1 Table), 2003 to 2015. Plots show the prediction when variables were fixed at a representative value (July for the year effect, and 2009 for the month effect). Dotted lines are 95% confidence intervals. (DOCX) [file pone.0172396.s006.docx]

S1 Fig.

**S1 Fig. Numbers of southern elephant seal (*Mirounga leonina*) mortalities in the Heard Island and McDonald Islands Patagonian toothfish fishery have increased over time, being most common during the austral winter months.** Predicted mortalities per 10^-6^ longline hooks set by year (upper panel) and by month (lower panel) from a generalised additive model of data reported for longline vessels operating within the Heard Island and McDonald Islands Exclusive Economic Zone (HIMI-EEZ, data from S1 Table), 2003 to 2015. Plots show the prediction when variables were fixed at a representative value (July for the year effect, and 2009 for the month effect). Dotted lines are 95% confidence intervals.
