# Supplementary material for: Southern elephant seals (Mirounga leonina Linn.) depredate toothfish longlines in the midnight zone
Source: PLoS One. 2017 Feb 24;12(2):e0172396. doi: 10.1371/journal.pone.0172396 (PMC5325274; doi:10.1371/journal.pone.0172396)
Supplement: S2 Fig — Blue dotted lines show the depths at which the southern elephant seal interactions were recorded. (PDF) [file pone.0172396.s007.pdf]

S2 Fig.

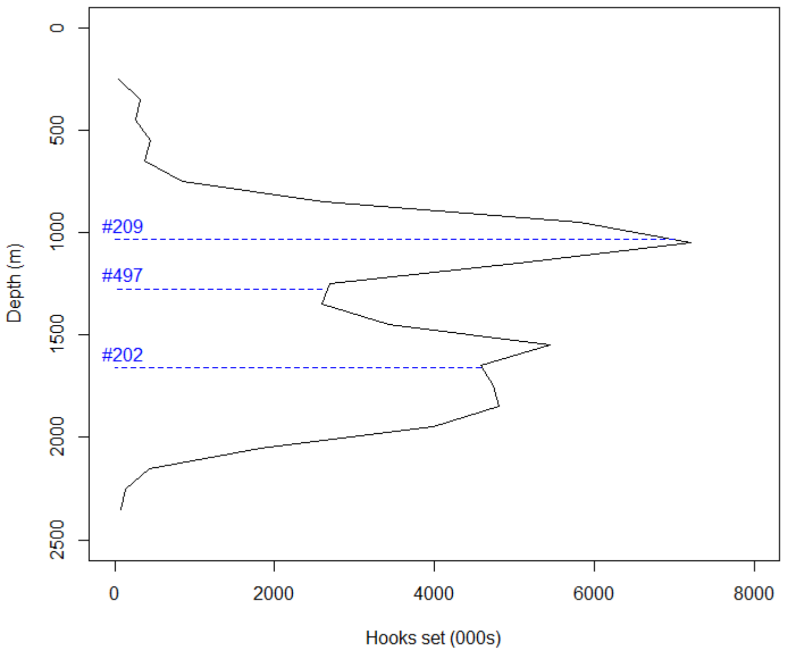


**S2 Figure. Depth distribution for Patagonian toothfish (*Dissostichus eleginoides*) longline fishing effort (number of hooks set) and three male southern elephant seals (*Mirounga leonina*) seen interacting with toothfish longlines within the Heard Island McDonald Islands Exclusive Economic Zone (HIMI-EEZ).** Blue dotted lines show the depths at which the southern elephant seal interactions were recorded.
